# Supplementary material for: The deubiquitinase OTUD1 regulates immunoglobulin production and proteasome inhibitor sensitivity in multiple myeloma
Source: Nat Commun. 2022 Nov 10;13:6820. doi: 10.1038/s41467-022-34654-2 (PMC9649770; doi:10.1038/s41467-022-34654-2)
Supplement: Supplementary file 1 — Supplementary Information [file 41467_2022_34654_MOESM1_ESM.pdf]

Supplementary Figure 1

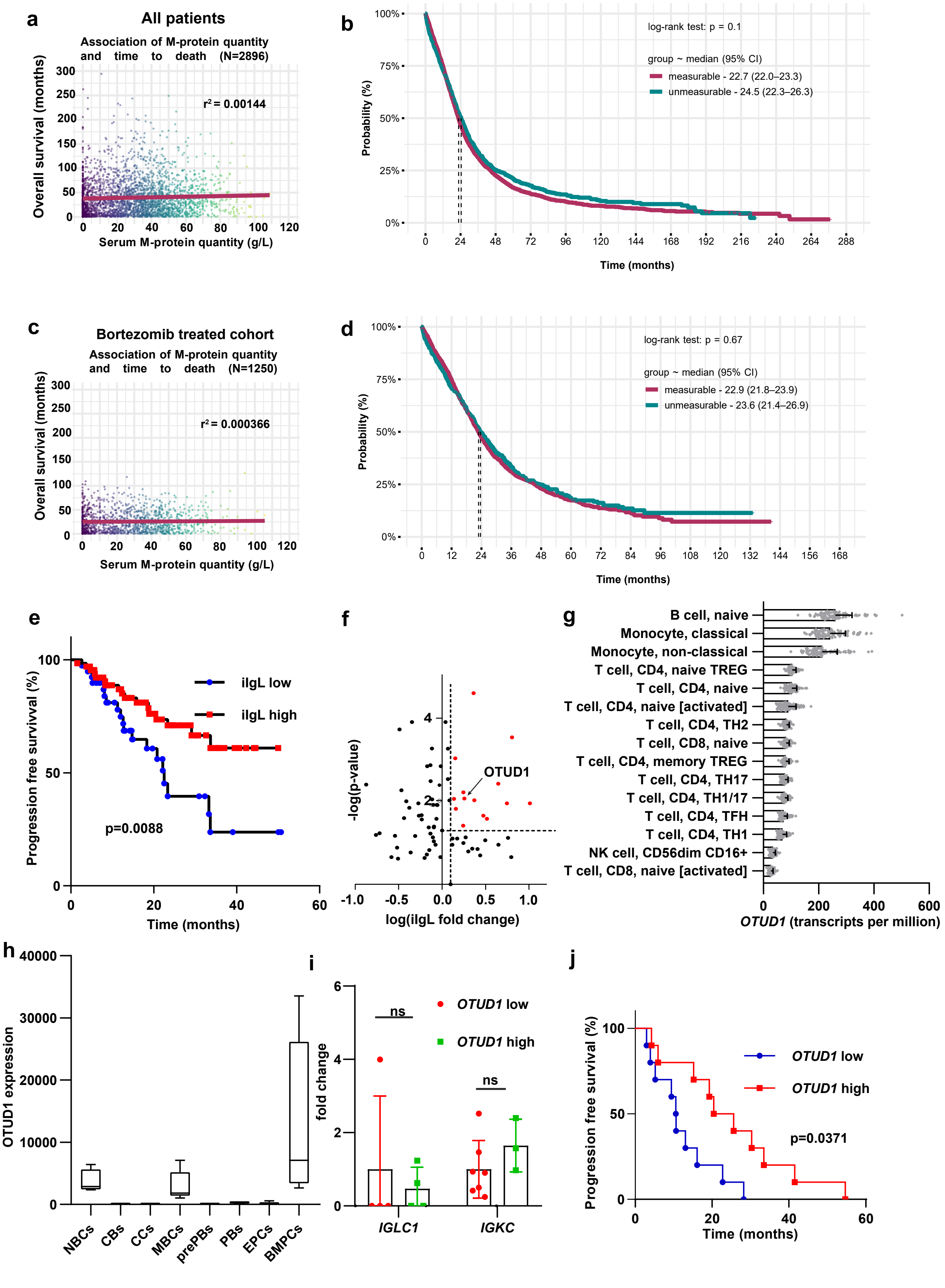

Supplementary Figure 1. Serum M-protein does not predict MM patient survival and OTUD1 is a unique plasma cell DUB. a Correlation analysis of serum M-protein with overall survival (OS) of newly diagnosed multiple myeloma (MM) patients regardless of the applied treatment (n=2896). **b** Progression-free survival (PFS) of MM patients divided into two groups: measurable and unmeasurable disease. Measurability of disease according to paraprotein is defined

as: serum M-protein quantity (g/l) > 10g/l or serum kappa free-light chains (FLC) quantity (mg/l) > 200 mg/l or serum lambda FLC quantity (mg/l) > 200 mg/l. Significance was compared using two-sided log-rank (Mantel-Cox) test (n=4424 for measurable, n=1054 for unmeasurable, p=0.1). **c** Correlation analysis of serum M-protein with OS of newly diagnosed MM patients receiving bortezomib in the first line of therapy (n=1250). **d** PFS of MM patients treated with bortezomib. Divided into two groups: measurable and unmeasurable disease. Measurability of disease according to paraprotein is defined as: serum M-protein quantity (g/l) > 10g/l or serum kappa FLC quantity (mg/l) > 200 mg/l or serum lambda FLC quantity (mg/l) > 200 mg/l. Significance was compared using two-sided log-rank (Mantel-Cox) test (n=2570 for measurable, n=589 for unmeasurable, p=0.67). **e** PFS of MM patients divided into two groups based on Flow cytometry analysis of iIgL in aberrant plasma cells. Significance was compared using two-sided log-rank (Mantel-Cox) test (n=40 for iIgL low, n=66 for iIgL high, p=0.0088). **f** Scatter plot representing combined results of CRISPR screen (RPMI8226 cells) and survival analysis (MM patients). For the screen, RPMI8226 cells stably expressing Cas9 were transduced with sgRNA targeting human deubiquitinase (DUB) genes (n=72, 3 sgRNA/gene) and iIgL content was analysed by flow cytometry. The influence of DUB expression on MM patient survival was determined from the gene expression dataset GSE4581. Significance was compared using log-rank test. Log(fold change) was determined as positive, if iIgL(sgDUB) / iIgL(sgcontrol) and hazard ratio were both >1 or both <1. -Log(pvalue) for each gene was calculated from the Kaplan–Meier analysis of MM patient OS. The threshold for fold change is 20% and 0.05 for p-value. **g** Expression of OTUD1 in different types of blood cells according to the DICE database. (NK cells, n=90; CD8 T cells, n=89; monocytes, n=91; TH17 cells, n=89; TH2 cells, n=89; T cell, CD4, naive [activated], n=88; B cell, naïve, n=91; Monocyte, non-classical, n=90; T cell, CD4, TFH, n=89; T cell, CD8, naive [activated], n=88; T cell, CD4, TH1/17, n=88; T cell, CD4, memory TREG, n=89; T cell, CD4, naïve, n=88; T cell, CD4, TH1, n=81; T cell, CD4, naive TREG, n=89.) Data are represented as mean ± SD. **h** Changes in the OTUD1 expression in the course of naïve B cells differentiation according to the GenomicScape tool. Abbreviations: naive B cells (NBC), centroblasts (CB), centrocytes (CC), memory B cells (MBC), preplasmablasts (prePB), plasmablasts (PB), early plasma cells (EPC), and bone marrow plasma cell (BMPC). (Number of biologically independent samples: Naïve B cells (n=5); Centroblasts (n=4); Centrocytes (n=4); Memory B cells (n=5); Preplasmablasts (n=5); Plasmablasts (n=5); Early plasma cells (n=5); Bone marrow plasma cells (n=5). The whiskers represent minimal and maximal values, the box extends from the 25th to 75th percentiles. **i** Expression of IGLC1 and IGKC was analysed by RT-PCR in OTUD1 low and OTUD1 high groups from **Fig. 1g**. Significance was compared using an two-tailed Student's t-test, ns = non-significant. Data are represented as mean ± SD. **j** PFS of MM patients from (**Fig. 1b**), divided into two groups based on median OTUD1 expression in aberrant plasma cells. Significance was compared using two-sided log-rank (Mantel-Cox) test (n=10 for OTUD1 low, n=10 for OTUD1 high, p=0.0371).

Supplementary Figure 2.

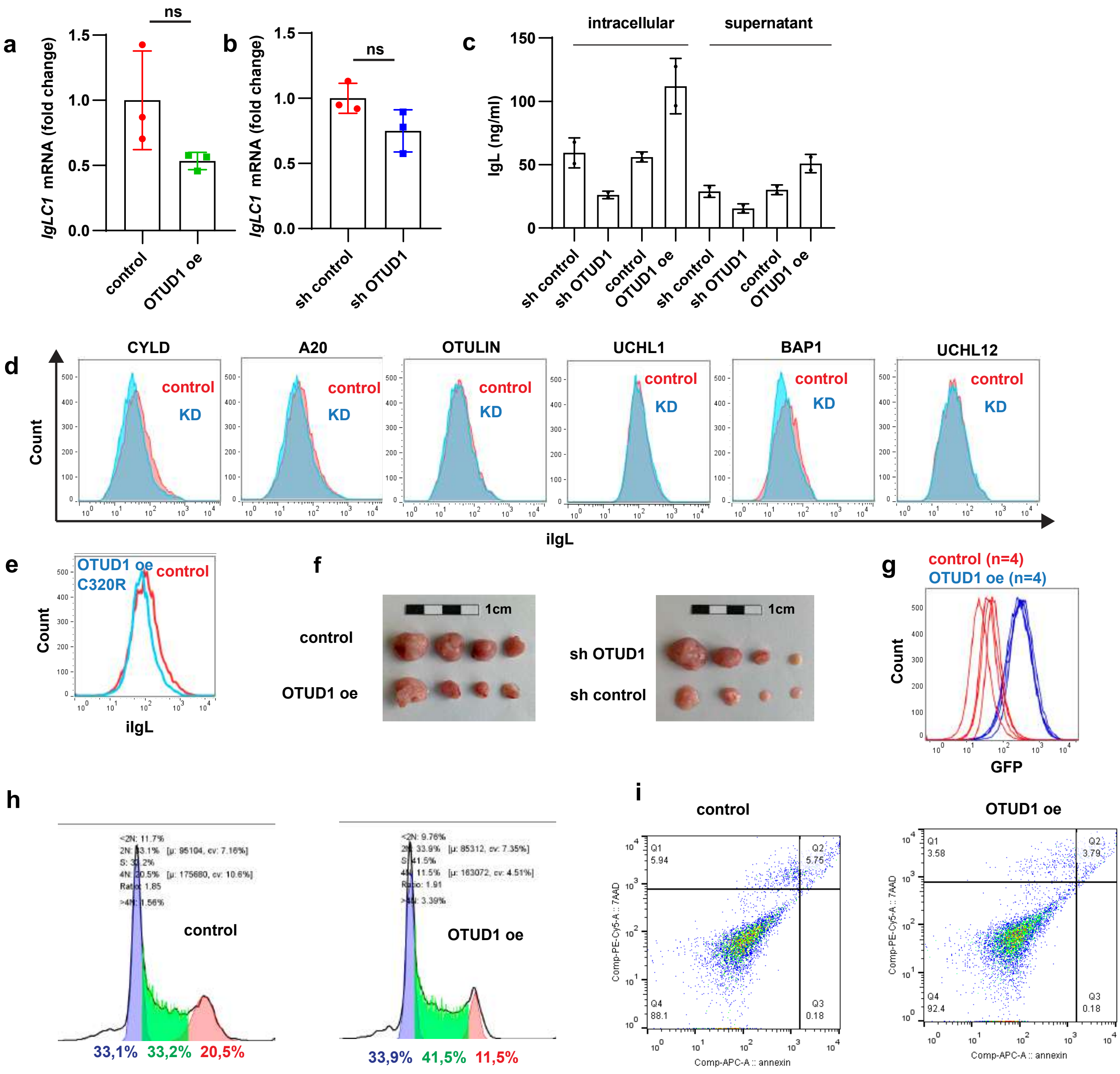

Supplementary Figure 2. OTUD1 catalytic activity is required for post-transcriptional regulation of Ig production in MM cells. a, b Expression of IGLC1 was analysed by RT-PCR in OTUD1 oe and sh OTUD1 RPMI8226 cells and normalized to the respective controls (n=3 independent experiments). Significance was compared using two-tailed t-test, ns = non-significant. Data are represented as mean ± SD. **c** IgL concentration was determined by ELISA in cell lysates or growth media of the respective RPMI8226 cell lines described in Fig. 2a, b. (n=2 independent experiments) Data are represented as mean ± SD. **d** iIgL content was analysed by intracellular staining and flow cytometry in cells with knock-down (KD) of specific DUB and their respective isogenic controls. **e** iIgL content was analysed by flow cytometry in RPMI8226 cells with doxycycline-inducible overexpression of OTUD1 catalytically inactive mutant (OTUD1 oe C320R) and compared to non-induced isogenic control. f Size of tumor xenografts 5 weeks after injection. g Control and OTUD1 oe mouse xenografts (n=4 for each group) were mechanically dissociated into single-cell suspension and GFP expression was analysed by flow cytometry. h Flow cytometry-based analysis of cell cycle profile in control and OTUD1 oe RPMI8226 cells. i AnexinV-stained control and OTUD1 oe RPMI8226 cells were analysed by flow cytometry.

Supplementary Figure 3.

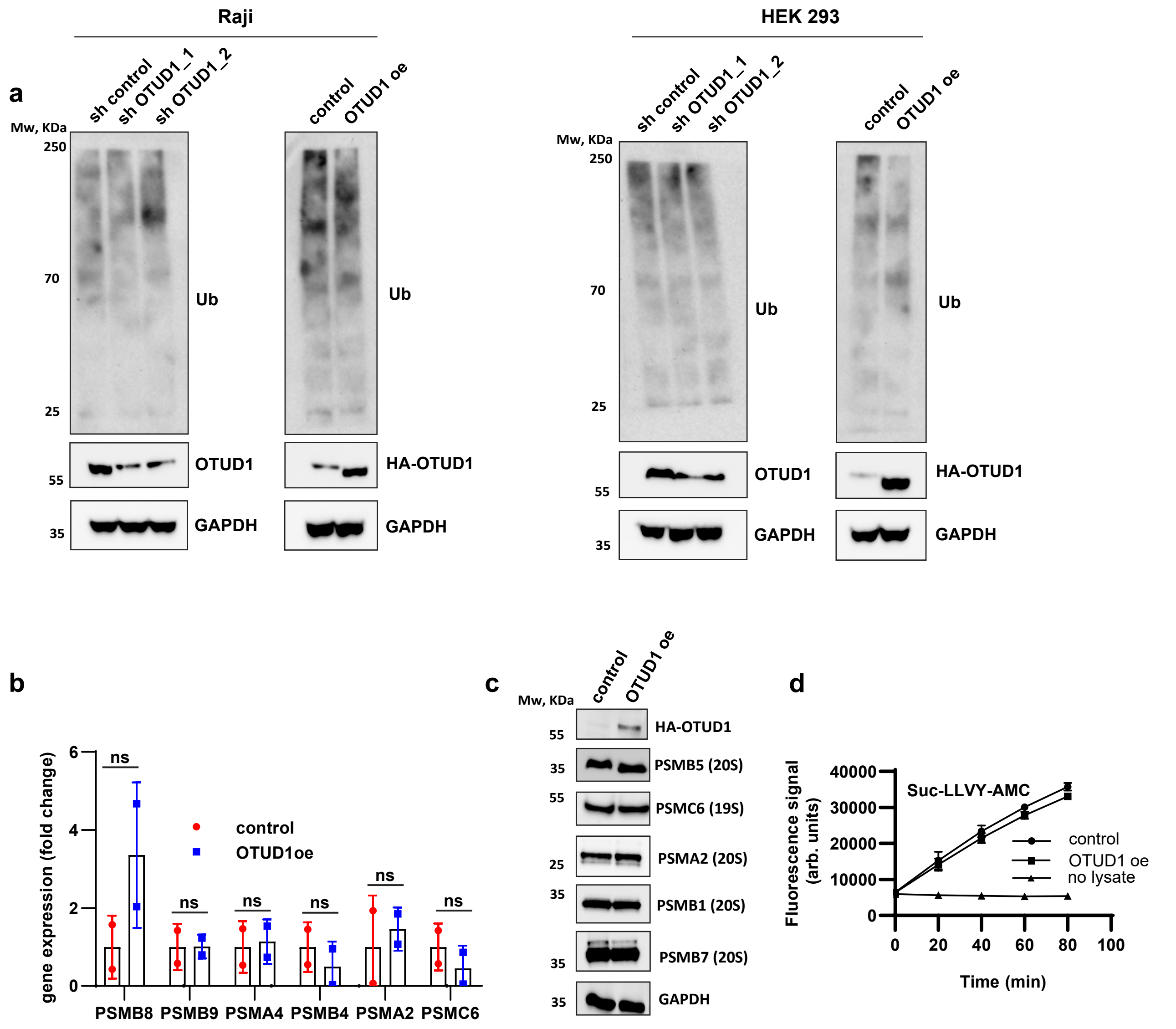

Supplementary Figure 3. OTUD1 does not affect expression and activity of proteasome but modules load on proteasome. a Total amount of ubiquitinated proteins in extracts from OTUD1 oe, and sh OTUD1 expressing Raji and HEK293 cells was analysed by western blotting. **b** PSMB8 and PSMB9 expression was analysed by RT-PCR in RPMI8226 with doxycycline-inducible OTUD1 overexpression (OTUD1 oe) and non-induced isogenic control cells (n=2 independent experiments). Significance was compared using two-tailed t-test, ns = non-significant. Data are represented as mean ± SD. **c** Western blot analysis of PSMB5 and PSMB6 in cell from (a). d Proteasome enzymatic activity was estimated in OTUD1 oe RPMI8226 cells by measuring the cleavage of fluorogenic substrate Suc-LLVY-AMC (n=2 independent experiments). Data are represented as mean ± SD.

Supplementary Figure 4.

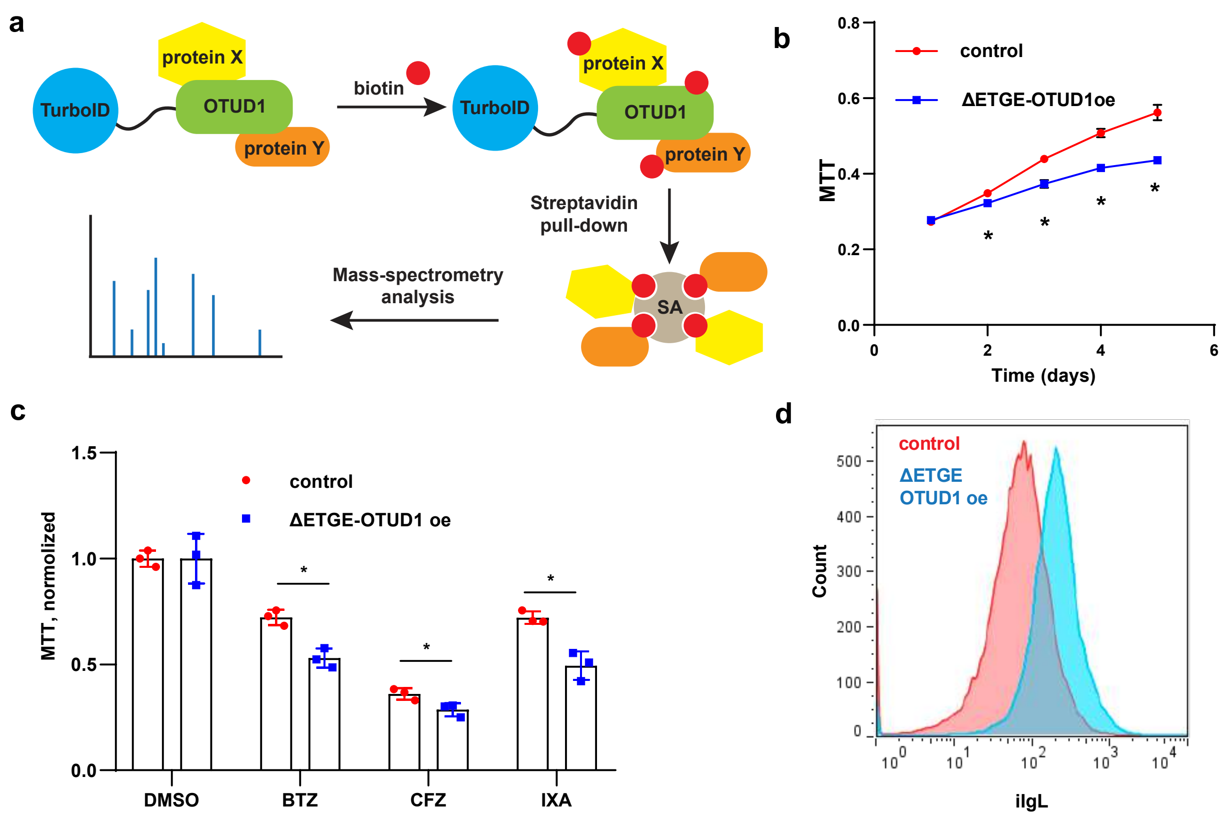

Supplementary Figure 4. KEAP1 binding is not required for OTUD1-controlled Ig production, PIs sensitivity and proliferation of MM cells. a Schematic representation of TurboID assay (SA = Streptavidin sepharose). **b** RPMI2886 cells with doxycycline-inducible expression of OTUD1 ΔETGE mutant (ΔETGE-OTUD1 oe) were plated for each time point, cell proliferation was estimated by MTT assay during 5 days and compared to the isogenic controls (n=3 independent experiments). Significance was compared using two-way ANOVA test (p=0.0033). Data are represented as mean ± SD. **c** RPMI2886 cells with inducible expression of OTUD1 ΔETGE were left treated with vehicle (DMSO) or with 10 nM bortezomib (BTZ), 5 nM carfilzomib (CFZ) or 10 nM ixazomib (IXA) for 16 h. Cell viability was estimated by MTT and normalized to the DMSO-treated sample (n=3 independent experiments). Significance was compared using two-tailed t-test (BTZ p=0.0046, CFZ p=0.0366, IXA p=0.0059) . Data are represented as mean ± SD. **d** iIgL content was analysed by flow cytometry in RPMI8226 cells with inducible expression of OTUD1 ΔETGE and compared to isogenic control (representative experiment, n=3).

Supplementary Figure 5.

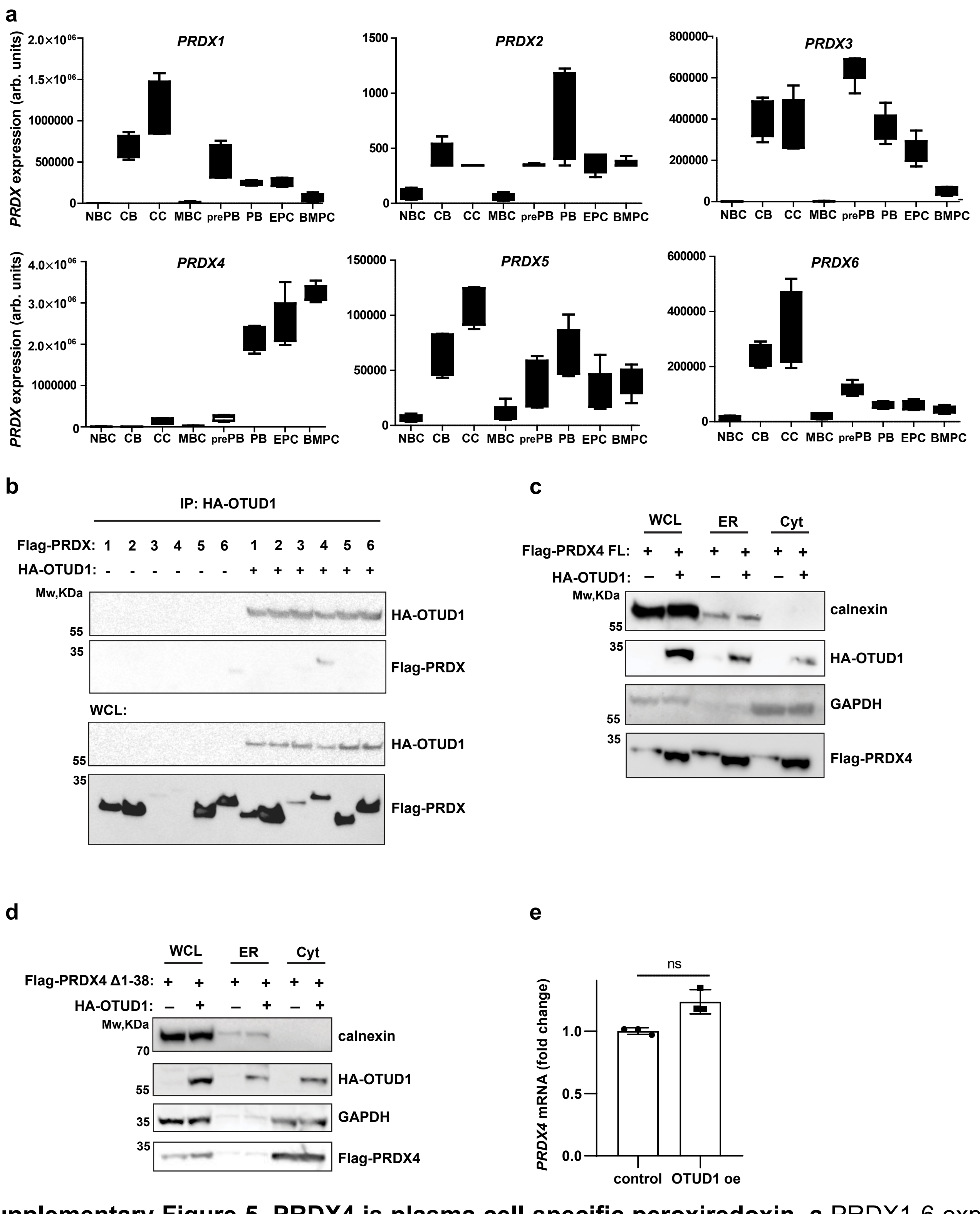

Supplementary Figure 5. PRDX4 is plasma cell-specific peroxiredoxin. a PRDX1-6 expression in the course of naïve B cells differentiation according to the GenomicScape tool. Abbreviations: naive B cells (NBC), centroblasts (CB), centrocytes (CC), memory B cells (MBC), preplasmablasts (prePB), plasmablasts (PB), early plasma cells (EPC), and bone marrow plasma cell (BMPC). (Number of biologically independent samples: Naïve B cells (n=5); Centroblasts (n=4); Centrocytes (n=4); Memory B cells (n=5); Preplasmablasts (n=5); Plasmablasts (n=5); Early plasma cells (n=5); Bone marrow plasma cells (n=5). The whiskers represent minimal and maximal values, the box extends from the 25th to 75th percentiles. **b** HA-tagged OTUD1 and Flag-tagged PRDX1-6 were expressed in HEK 293T cells and immunoprecipitated using

anti-Flag agarose resin. Western blot analysis was used to detect co-immunoprecipitated proteins and protein expression in whole cell lysate (WCL). **c, d** The presence of HA-tagged OTUD1 and Flag-tagged FL PRDX4 (d) and Δ1-38 PRDX4 in different subcellular fractions was analysed by immunoblotting using anti-Flag, anti-HA, calnexin and GAPDH antibodies. **e** PRDX4 expression was analysed by RT-PCR in RPMI8226 with doxycycline-inducible OTUD1 expression (OTUD1 oe) and isogenic control cells (n=3 independent experiments). Significance was compared using twotailed t-test, ns = non-significant. Data are represented as mean ± SD.

Supplementary figure 6

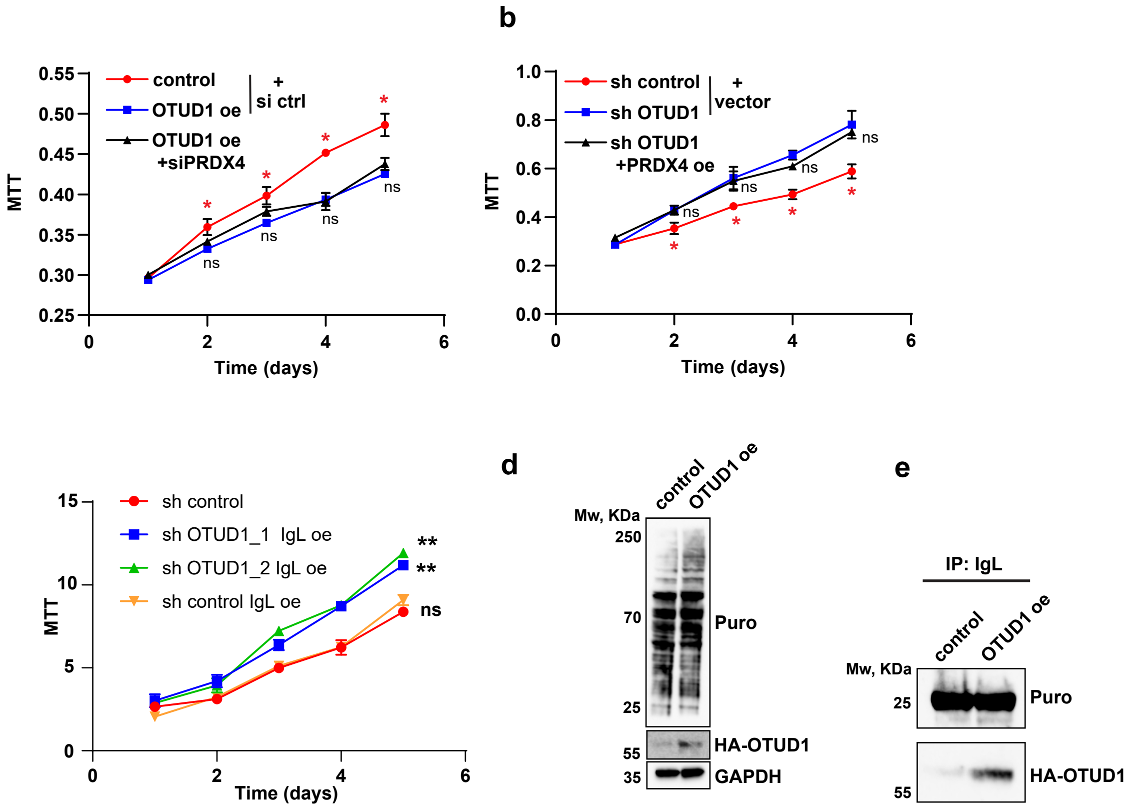

Supplementary Figure 6. OTUD1-mediated myeloma proliferation is not dependent on PRDX4 and OTUD1 does not affect translation initiation. **a,b** 10^4 of OTUD1 oe (**a**) or shOTUD1 (**a**) RPMI8226 cells with and without transfection of siPRDX4 (**b**) or with or without PRDX4 oe (**b**) were plated for each time point. Cell proliferation was estimated by MTT assay during 5 days and compared to the respective isogenic controls. Significance was compared using the two-way ANOVA (in **a**: OTUD1 oe p=0.0315, OTUD1 oe + siPRDX4 p=0.0787; in **b**: shOTUD1 p=0.0331, shOTUD1 + PRDX4 oe p=0.0570), ns = non-significant. Data are represented as mean ± SD from 3 biological replicates. **c** 104 of sh control, sh OTUD1 oe and siIgL-transfected RPMI8226 cells were plated for each time point. Cell proliferation was estimated by MTT assay during 5 days and compared to the respective isogenic controls (n=3 independent experiments). Significance was compared using two-way ANOVA test (sh control IgL oe p=0.0628, shOTUD1_1 IgL oe p=0.0068, shOTUD1_2 IgL oe p=0.0036). Data are represented as mean ± SD. **d, e** Overall level of translation initiation was assessed by puromycin incorporation SUnSET assay. Whole cell lysates from RPMI8226 cells expressing control vector or OTUD1 were visualized using anti-Puromycin antibody. b IgL was immunoprecipitated from cell lysates treated as in (**d**) and immunoblotted using anti-puromycin antibody. HA-OTUD1 depicts expression of OTUD1 in whole cell lysate.

Supplementary Figure 7

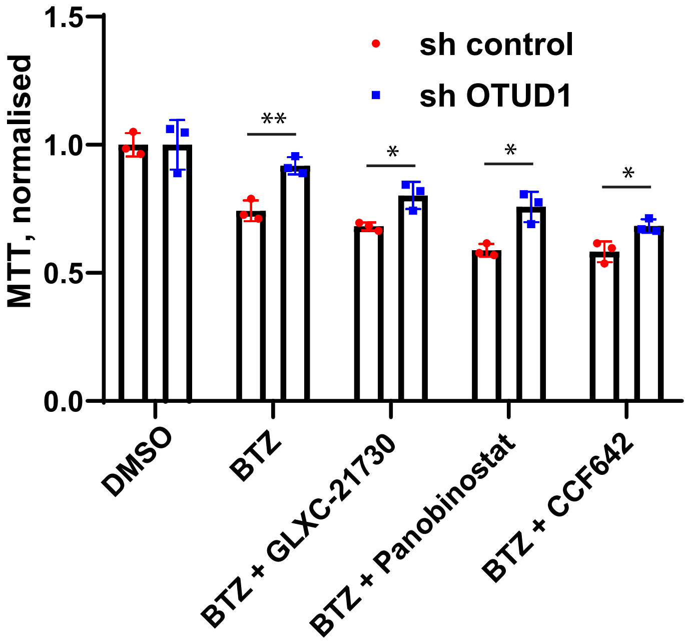

Supplementary Figure 7. Oxidative stress-inducing drugs fail to overcome PIs resistance in iIgL low MM cells. a RPMI8226 cells stably expressing sh OTUD1 were treated with 10 nM bortezomib (BTZ) alone or 10 nM BTZ in combination with 1 µM NADPH inhibitor GLX7013114, 10 nM HDAC inhibitor Panobinostat or 10 µM PDI inhibitor CCF642 for 16 h. Cell viability was estimated by MTT and normalized to the vehicle (DMSO)-treated sample (n=3 independent experiments). Significance was compared using two-tailed t-test (BTZ p=0.0046, BTZ + GLXC-21730 p=0.0192, BTZ + Panobinostat p=0.0104, BTZ + CCF642 p=0.0236). Data are represented as mean ±SD.

Supplementary Figure 8

a

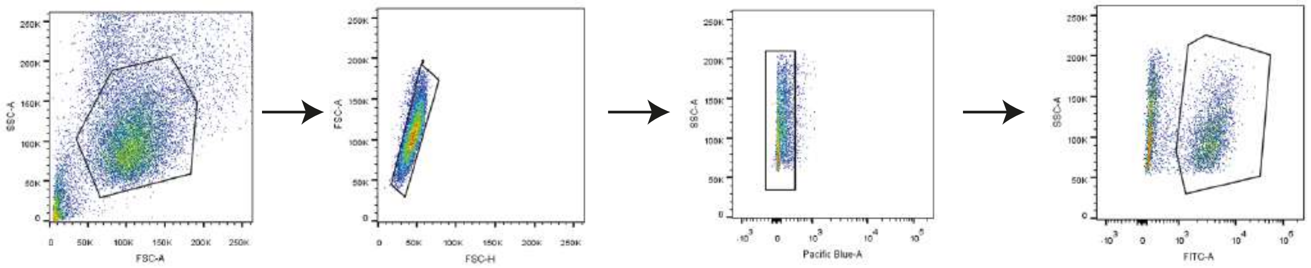

b

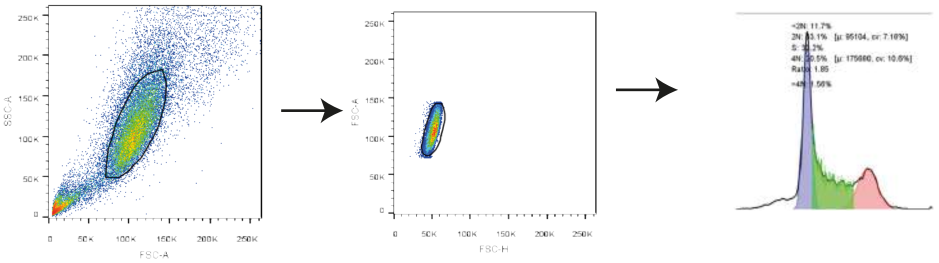

c

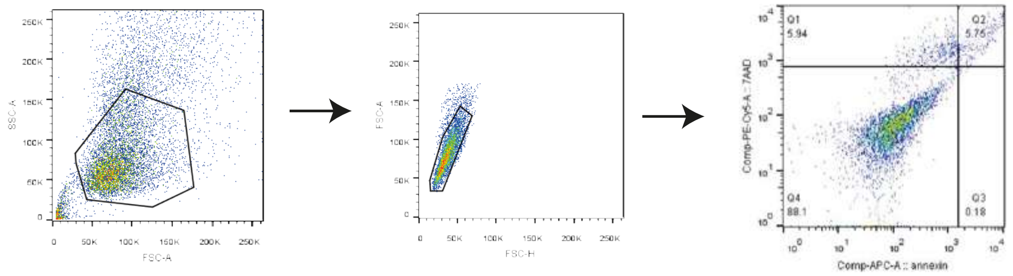

Supplementary Figure 8. Gating strategy used to analyse the results of flow cytometry experiments. a Gating strategy used for Fig. 2a,2d-e,2i,2k-m 3h, 6c-d, 7b, supplementary figure 1f, supplementary figure 2d-e, supplementary figure 2g, supplementary figure 4d. 1) Gating on morphologically homogenous cell population based on forward and side scatter. 2) Excluding cell duplets by gating on FSC-A vs FSC-H scatterplot. 3) Excluding dead cells by gating negative population in SYTOX™ Blue Dead Cell Staining. The resulted population was used to analyse the fluorescence intensity after of anti-Iglc anti-body or Bodipy staining (except 2a, d, 3h, 6c.) 4) In experiments with inducible OTUD1 overexpression (2a, d, 3h, 6c) GFP positive cells were gated. The resulted population was used to analyse the fluorescence intensity after anti-Iglc antibody or Bodipy staining. **b** Gating strategy in cell cycle analysis (supplementary figure 2h). 1) Gating on morphologically homogenous cell population based on forward and side scatter. 2) Excluding cell duplets by gating on FSC-A vs FSC-H scatterplot. 3) Analyse DNA content using the Watson model. **c** Gating strategy in analysis of cell viability (supplementary figure 2i) 1) Gating on morphologically homogenous cell population based on forward and side scatter. 2) Excluding cell duplets by gating on FSC-A vs FSC-H scatterplot. 3) Analysis of 7AAD and Annexin-V costaining

Supplementary Table 1.

correlation analysis of serum M-protein with OS and PFS (Supplementary Fig. 1a, b).

	**Patients in 1st line (n=5478)**
**Sex**, n (%)	n=5478
male	2867 (52.3%)
female	2611 (47.7%)
**Age**	n=5478
mean(sd)	65.8 (10.6)
median (5–95% quantile)	66.0 (47.0–82.0)
min.–max.	17.0–95.0
**ISS stage**, n (%)	n=5254
stage I	1651 (31.4%)
stage II	1767 (33.6%)
stage III	1836 (34.9%)
missing	224
**M-protein type**, n (%)	n=5462
IgG	3270 (59.9%)
IgA	1153 (21.1%)
LC only	819 (15.0%)
Non-secretory	85 (1.6%)
Biclonal	64 (1.2%)
IgM	35 (0.6%)
IgD	32 (0.6%)
Triclonal	3 (0.1%)
IgE	1 (0.0%)
missing	16

ISS, International staging system

Supplementary Table 2.

correlation analysis of serum M-protein with OS and PFS (Supplementary Fig. 1c, d).

	**Patients in 1st line with Bortezomib (n=3519)**
**Sex**, n (%)	n=3519
male	1690 (53.5%)
female	1469 (46.5%)
**Age**	n=3159
mean(sd)	66.0 (10.1)
median (5–95% quantile)	67.0 (47.0–81.0)
min.–max.	(17.0–91.0)
**ISS stage**, n (%)	n=3089
stage I	844 (27.3%)
stage II	1005 (32.5%)
stage III	1240 (40.1%)
missing	70
**M-protein type**, n (%)	n=3145
IgG	1817 (57.8%)
IgA	658 (20.9%)
LC only	555 (17.6%)
Biclonal	43 (1.4%)
Non-secretory	27 (0.9%)
IgM	22 (0.7%)
IgD	22 (0.7%)
Triclonal	1 (0.0%)
missing	14

ISS, International staging system

Supplementary Table 3.

correlation of iIgL with PFS, and survival analysis (Fig. 1a-c and Supplementary Fig. 1e).

	**Patients (n=142)**
**Sex**, n (%)	n=142
male	71 (50%)
female	71 (50%)
**Age**	n=142
mean(sd)	67.3 (10)
median (5–95% quantile)	68.25 (49.63-81.08)
min.–max.	42-90
**ISS stage**, n (%)	n=140
stage I	47 (33.6%)
stage II	57 (40.7%)
stage III	36 (25.7%)
**LDH**	n=134
mean (sd)	3.3 (1.6)
normal (≤5)	125 (93.3%)
LC only	9 (6,7%)

ISS, International staging system

Supplementary Table 4. Mulvariate Cox hazard model of basic clinical characteristics of newly diagnosed patients included in the correlation analysis of iIgL with PFS (Fig. 1b).

Variable		Hazard ratio	95% Confidence interval	P-value
**Intracellular Ig**				
	high (N=27)	1		
	low (N=59)	2.65	1.21- 5.8	0.014
**ISS**				
	Stage 1 (N=22)	1		
	Stage 2 (N=38)	1.24	0.55-2.8	0.603
	Stage 3 (N=25)	2.71	1.15-6.4	0.014
**Age**				
	≤65 (N=48)	0.84	0.47-1.5	0.565
	>65 (N=38)	1		
**Sex**				
	Male (N=38)	1		
	Female (N=45)	0.97	0.54-1.7	0.568
**LDH**				
	Normal (N=72)	1		
	Elevated (N=11)	1.84	0.85-4	0.124

Supplementary Table 5. DUBs affecting Ig production and MM patient survival (Supplementary Fig. 1f).

Name	log(fold change)	-log (p)
USP5	0.13703569	2.065502
OTUD6B	0.15308	3.045757
TRABD	0.158608	1.823909
USP47	0.24361985	1.420216
FAM105A	0.245427	2.222573
OTUD1	0.25672958	2.075721
USP35	0.36333803	4.619789
USPL1	0.36993651	2.031517
USP42	0.47540634	1.657577
USP52	0.5160958	1.585027
USP12	0.64690744	2.431798
USP40	0.74640377	1.958607
USP33	0.80495076	3.552842
USP18	1.01047212	1.958607
