# Supplementary material for: The deubiquitinase OTUD1 regulates immunoglobulin production and proteasome inhibitor sensitivity in multiple myeloma
Source: Nat Commun. 2022 Nov 10;13:6820. doi: 10.1038/s41467-022-34654-2 (PMC9649770; doi:10.1038/s41467-022-34654-2)
Supplement: Supplementary file 3 — Description of Additional Supplementary Files [file 41467_2022_34654_MOESM3_ESM.pdf]

Description of Additional Supplementary Files

Supplementary Data 1: Genes uniquely expressed in plasma cells compare to other B-cell developmental stages

Supplementary Data 2: Proteomic analysis of OTUD1 interactors

Supplementary Data 3: Oligonucleotides used in the study
