# Supplementary material for: The deubiquitinase OTUD1 regulates immunoglobulin production and proteasome inhibitor sensitivity in multiple myeloma
Source: Nat Commun. 2022 Nov 10;13:6820. doi: 10.1038/s41467-022-34654-2 (PMC9649770; doi:10.1038/s41467-022-34654-2)
Supplement: Supplementary file 7 — Reporting Summary [file 41467_2022_34654_MOESM7_ESM.pdf]

nature portfolio

Corresponding author(s): Michal Simicek

Last updated by author(s): Oct 12, 2022

Reporting Summary

Nature Portfolio wishes to improve the reproducibility of the work that we publish. This form provides structure for consistency and transparency in reporting. For further information on Nature Portfolio policies, see our Editorial Policies and the Editorial Policy Checklist.

Statistics

For all statistical analyses, confirm that the following items are present in the figure legend, table legend, main text, or Methods section.

n/a	Confirmed	
☐	☒	The exact sample size (*n*) for each experimental group/condition, given as a discrete number and unit of measurement
☐	☒	A statement on whether measurements were taken from distinct samples or whether the same sample was measured repeatedly
☐	☒	The statistical test(s) used AND whether they are one- or two-sided<br>*Only common tests should be described solely by name; describe more complex techniques in the Methods section.*
☒	☐	A description of all covariates tested
☒	☐	A description of any assumptions or corrections, such as tests of normality and adjustment for multiple comparisons
☐	☒	A full description of the statistical parameters including central tendency (e.g. means) or other basic estimates (e.g. regression coefficient) AND variation (e.g. standard deviation) or associated estimates of uncertainty (e.g. confidence intervals)
☐	☒	For null hypothesis testing, the test statistic (e.g. *F*, *t*, *r*) with confidence intervals, effect sizes, degrees of freedom and *P* value noted<br>*Give P values as exact values whenever suitable.*
☒	☐	For Bayesian analysis, information on the choice of priors and Markov chain Monte Carlo settings
☒	☐	For hierarchical and complex designs, identification of the appropriate level for tests and full reporting of outcomes
☐	☒	Estimates of effect sizes (e.g. Cohen's *d*, Pearson's *r*), indicating how they were calculated

Our web collection on statistics for biologists contains articles on many of the points above.

Software and code

Policy information about availability of computer code

Data collection	All qPCR data was collected using StepOne™ Software v2.3 (available at https://www.thermofisher.com/cz/en/home/technical-resources/software-downloads/StepOne-and-StepOnePlus-Real-Time-PCR-System.html).<br>Flow cytometry data was acquired by BD FACSDiva™ Software v9.0 and CytExpert software v2.4.<br>In vivo bioluminescence data was obtained by Bruker MI SE 7.2 (https://www.bruker.com/protected/en/services/software-downloads/molecular-imaging.html).
Data analysis	Plots were generated and statistically analyzed in GraphPad Prism 5.<br>Flow cytometry data was analyzed in FlowJo v10.<br>In vivo bioluminescence data were analysed in Bruker MI SE 7.2 (https://www.bruker.com/protected/en/services/software-downloads/molecular-imaging.html).<br>Mass spectrometry data analysis was performed using MaxQuant 1.6.3.4 and Perseus 1.6.1.3 software.<br>Densitometry analysis of immunoblots was perfomed using ImageJ v1.49 (National Institutes of Health).<br>Multivariate Cox proportional hazard model was computed using R 4.0.3 and survival v3.2.11 package.<br>Survival analysis was statistically evaluated in the SPSS software (IBM Corp. Released 2017. IBM SPSS Statistics for Windows, Version 25.0.0.1 Armonk, NY: IBM Corp.) and software R version 4.0.1. (www.r-project.org).<br>Cubic spline analysis was performed using rms v6.2-0 R package and R v4.0.5.

For manuscripts utilizing custom algorithms or software that are central to the research but not yet described in published literature, software must be made available to editors and reviewers. We strongly encourage code deposition in a community repository (e.g. GitHub). See the Nature Portfolio guidelines for submitting code & software for further information.

Data

Policy information about availability of data

All manuscripts must include a data availability statement. This statement should provide the following information, where applicable:

- Accession codes, unique identifiers, or web links for publicly available datasets
- A description of any restrictions on data availability
- For clinical datasets or third party data, please ensure that the statement adheres to our policy

The raw data of related to the patient cohort are deposited in the Czech Registry of Monoclonal Gammopathies and are available under restricted access, access can be obtained upon request. The raw proteomic data were deposited in the PRIDE database under accession number: PXD037309. Microarray and survival data for MM patients from Goswami CP et al., 2013 and Myeloma Institute for Research and Therapy, Donna D. and Donald M. Lambert Laboratory of Myeloma Genetics are publicly available from GEO database under accession numbers GSE2658 and GSE4581 respectively. Gene expression data in B cell lineage from Jourdan et al., 2014 is available at http://www.genomicscape.com/microarray/data_management.php?view=2. Gene expression in human immune cells data from Benjamin J. Schmiedel et al., 2018 is available at https://dice-database.org/landing. The remaining data are available within the Article, Supplementary Information file and Source Data file.

Human research participants

Policy information about studies involving human research participants and Sex and Gender in Research.

Reporting on sex and gender	Findings in this study are not related to one sex.
Population characteristics	The study included only patients diagnosed with multiple myeloma. The clinical characteristics are described in the Supplementary Table 1 and 2.
Recruitment	Only patients diagnosed with multiple myeloma were included in the study.
Ethics oversight	All patients gave a written informed consent before sample collection. The bone marrow collection and the study protocol were approved by the Ethical Committee of the University Hospital Ostrava. The bone marrow aspirates used in the study were stored in Biobank of the University Hospital Ostrava.

Note that full information on the approval of the study protocol must also be provided in the manuscript.

Field-specific reporting

Please select the one below that is the best fit for your research. If you are not sure, read the appropriate sections before making your selection.

☒ Life sciences ☐ Behavioural & social sciences ☐ Ecological, evolutionary & environmental sciences

For a reference copy of the document with all sections, see nature.com/documents/nr-reporting-summary-flat.pdf

Life sciences study design

All studies must disclose on these points even when the disclosure is negative.

Sample size	For experiments with patients biological materials sample size was not precalculated due to limited by availability of bio-material.<br>In vivo experiments were performed with 4 mice in each group based on previous studies using same cell line for xenografts (Berahovich et at. 2017, PMID: 30208593).
Data exclusions	No data was excluded in the analyses.
Replication	All experiments were performed in at least 2 or more biological replicates. Experiments with patients samples were performed without biological replicates due to limited source of materials. No replicates were excluded from this study. All replicates displayed effects similar to those presented in the manuscript.
Randomization	Mice for in vivo experiments were randomly divided into two groups and recieved either WT or KD cells as xenograft. All additional experiments were performed in blinding manner to ensure no bias in subsequent analysis.
Blinding	All experiments were performed in blinded manner manner to ensure no bias in subsequent analysis. No clinical data about patients was known prior qPCR, ELISA and WB analysis.

Reporting for specific materials, systems and methods

We require information from authors about some types of materials, experimental systems and methods used in many studies. Here, indicate whether each material, system or method listed is relevant to your study. If you are not sure if a list item applies to your research, read the appropriate section before selecting a response.

Materials & experimental systems

n/a	Involved in the study
☐	☒ Antibodies
☐	☒ Eukaryotic cell lines
☒	☐ Palaeontology and archaeology
☐	☒ Animals and other organisms
☒	☐ Clinical data
☒	☐ Dual use research of concern

Methods

n/a	Involved in the study
☒	☐ ChIP-seq
☐	☒ Flow cytometry
☒	☐ MRI-based neuroimaging

Antibodies

Antibodies used

Mouse monoclonal anti-ß-Actin, clone 8H10D10, Cell Signaling Technology , Cat#: 3700S
Mouse monoclonal anti-FLAG, clone M2, Sigma Aldrich , Cat#: F1804
Mouse monoclonal anti-GAPDH, clone D4C6R, Cell Signaling Technology , Cat#: 97166S
Rat monoclonal anti-HA, clone 3F10, Roche, Cat#: 11867423001
Mouse monoclonal anti-Puromycin, clone 12D10, Sigma Aldrich , Cat#: MABE343
Mouse polyclonal anti-Human IgG (H+L), Sigma Aldrich , Cat#: SAB3701329
Polyclonal anti-Human Ig lambda antibody labelled with APC-C750 , Cytognos, S.L. , Cat#: CYT-LAC750
Rabbit monoclonal anti-KEAP1, D6B12, Cell Signaling Technology , Cat#: 8047S
Rabbit polyclonal anti-OTUD1 sera, Moravian Biotechnology , N/A
Mouse monoclonal anti-PolyHistidine, clone HIS1, Sigma Aldrich , Cat#: H1029
Mouse monoclonal anti-PRDX4, Proteintech , Cat#: 60286-1-Ig
Rabbit polyclonal anti-PRDX4, Proteintech , Cat#: 10703-1-AP
Rabbit polyclonal anti-PSMC5, Bethyl Laboratories , Cat#: A303-825A
Mouse monoclonal anti-Ubiquitin, clone P4D1, Cell Signaling Technology , Cat#: 3936S
Rabbit monoclonal anti-phospho-eIF2a (Ser51), clone D9G8, Cell Signaling Technology , Cat#: 3398
Rabbit monoclonal anti-ATF6, clone D4Z8V, Cell Signaling Technology , Cat#: 65880S
Mouse monoclonal anti-calnexin, clone E10, Santa Cruz, Cat#: sc-46669
Rabbit polyclonal anti-PDIA6, Bethyl, Cat#: A304-519A
Rabbit monoclonal anti-PCNA, clone D3H8P, Cell Signaling Technology , Cat#: 13110S
Rabbit polyclonal anti-PSMB5, Proteintech , Cat#: 19178-1-AP
Rabbit polyclonal anti-PSMC6, Bethyl, Cat#: A303-825A
Mouse monoclonal anti-PSMA2, clone B4, Santa Cruz, Cat#: sc-377148
Mouse monoclonal anti-PSMB1, clone D9, Santa Cruz, Cat#: sc-374405
Mouse monoclonal anti-PSMB7, clone H3, Santa Cruz, Cat#: sc-365725
Goat polyclonal HRP-conjugated anti-rabbit, Jackson ImmunoResearch, Cat#: 115-035-144
Goat polyclonal HRP-conjugated anti-mouse, Jackson ImmunoResearch, Cat#: 115-035-146
Goat polyclonal HRP-conjugated anti-rat IgG, Cytiva, Cat#:NA935

Validation

All commercially available antibodies have been tested by manufacturer for the use in western blot, immunoprecipitation or flow cytometry and applied accordingly in the study.
Custom anti-OTUD1 polyclonal rabbit sera was validated for use in western blotting on lysates from human cell lines with wild type and knock out OTUD1 and using purified recombinant human full lenght OTUD1 protein.

Eukaryotic cell lines

Policy information about cell lines and Sex and Gender in Research

Cell line source(s)

Human RPMI8226 cell line,ATCC, Cat#: CCL-155
Human MM.1S cell line, ATCC, Cat#: CRL-2974
Human JJN-3 cell line, DSMZ, Cat#: ACC 541
Human SK-MM-2 cell line, DSMZ, Cat#: ACC 430
Human 293FT cell line, Invitrogen, Cat#: R70007
Human HEK 293 cell line, ATCC, Cat#: CRL-1573

Authentication

Cell lines were not authenticated after receiving from ATCC, DSMZ or Invitrogen. For all cell lines-involving experiments, only low passage number cells (n<10) were used.

Mycoplasma contamination

Cell lines were regularly tested negative for mycoplasma contamination.

Commonly misidentified lines
(See ICLAC register)

None of the used cell lines is present in the ICLAC register.

Animals and other research organisms

Policy information about studies involving animals; ARRIVE guidelines recommended for reporting animal research, and Sex and Gender in Research

Laboratory animals	Female SCID Mice CB17/Icr-Prkdc-scid/IcrIcoCrl, 7 weeks old. Mice were hold in IVC cages at ambient temperature (22°C), 50% humidity with 12h light/dark cycle.
Wild animals	The study did not involve wild animals.
Reporting on sex	The results does not apply to specific sex.
Field-collected samples	No field-collected samples were used in this study.
Ethics oversight	All animal experiments were approved by the Animal Ethics Committee of the Faculty of Medicine, Ostrava University and the Animal Ethic Board of the Ministry of Education, Youth and Sport of the Czech Republic n. MSMT-4072/2021-3.

Note that full information on the approval of the study protocol must also be provided in the manuscript.

Flow Cytometry

Plots

Confirm that:

- [x] The axis labels state the marker and fluorochrome used (e.g. CD4-FITC).
- [x] The axis scales are clearly visible. Include numbers along axes only for bottom left plot of group (a 'group' is an analysis of identical markers).
- [x] All plots are contour plots with outliers or pseudocolor plots.
- [x] A numerical value for number of cells or percentage (with statistics) is provided.

Methodology

Sample preparation	Bone marrow aspirates were diluted in the Red Blood Cell lysis buffer (155 mM NH4Cl, 12 mM NaHCO3, 0.1 mM EDTA; 1 ml to 10 ratio) and incubated at room temperature for 15 min. Aberrant plasma cell phenotype was determined according to EuroFlow protocol (stained for CD38, CD138, CD45, CD56, CD117, CD27), cells were sorted and processed immediately.<br>For the in vivo experiment after mice sacrifice, tumor xenografts were mechanically dissociated into single-cell suspension and iIgL content and GFP expression were analyzed by flow cytometry.
Instrument	Flow cytometry experiments were performed on BD FACSAria II and CytoFLEX S.
Software	BD FACSDiva™ Software v9.0 and CytExpert v2.4 were used for data collection, FlowJo v10 was used for data analysis.
Cell population abundance	Aberrant plasma cells were determined by staining with antibody panel (CD38, CD138, CD45, CD56, CD117, CD27) according to the EuroFlow protocol. Infiltration for different samples varied from 8 to 87%.
Gating strategy	For flow cytometry of myeloma cells following gating strategy was used:<br>Homogenous in size and granularity cell population was gated on FSC-A/SSC-A plot to separate from cell debris, then on FSC-A/FSC-H cells were gated to exclude duplets. Then dead cells were excluded according to Live/Dead fixable staining. For OTUD1 OE cells, GFP positive cells were gated.

- [x] Tick this box to confirm that a figure exemplifying the gating strategy is provided in the Supplementary Information.
